# One‐Third Tubular Plate Remains a Clinically Good Option in Danis‐Weber Type B Distal Fibular Fracture Fixation

**DOI:** 10.1111/os.13160

**Published:** 2021-10-27

**Authors:** Jae Hoon Ahn, Sung Hyun Cho, Mingi Jeong, Yoon‐Chung Kim

**Affiliations:** ^1^ Department of Orthopaedic Surgery Seoul St. Mary's Hospital College of Medicine, The Catholic University of Korea Seoul Korea; ^2^ Department of Orthopaedic Surgery St. Vincent's Hospital, College of Medicine, The Catholic University of Korea Seoul Korea

**Keywords:** Clinical comparison, Danis‐Weber type B, Distal fibular fracture, Locking plate, One‐third tubular plate

## Abstract

**Objective:**

To compare the clinical outcomes of locking plate (LP) and non‐locking one‐third tubular plate (TP) fixation, and to provide guidance on plate selection for Danis‐Weber type B distal fibular fracture treatment.

**Methods:**

In total, 83 patients who underwent plate fixation for Danis‐Weber type B distal fibular fractures between March 2013 and July 2018 were retrospectively reviewed: 41 (49.0%) received LPs and 42 (51.0%) received TPs. Patients' demographic data, follow‐up durations, the proportion of comminuted fractures, and ankle range of motion were investigated. The American Orthopaedic Foot and Ankle Society (AOFAS) ankle‐hindfoot scale, Karlsson scale, Foot and Ankle Ability Measure (FAAM), and Lower Extremity Functional Scale (LEFS) scores were assessed. The radiographic union progression and implant removal time were evaluated, along with postoperative complications. Data from the LP and TP groups were compared statistically.

**Results:**

The mean patient ages were 53.3 ± 17.5 years (range, 16–80 years) and 47.6 ± 17.0 years (range, 14–68 years) in the LP and TP groups, respectively (*P >* 0.05). The gender distribution did not differ significantly between groups (*P >* 0.05). Other demographic data also did not differ significantly between groups (*P >* 0.05). The mean follow‐up durations were 16.8 ± 7.7 months (range, 13.0–19.0 months) in the LP group and 16.1 ± 6.2 months (range, 12.0–20.0 months) in the TP group (*P >* 0.05). Comminuted fractures were observed in 18 of 41 (43.9%) patients with LP and 10 of 42 (23.8%) patients with TP (*P >* 0.05). Forward bending ankle dorsiflexion was possible at the final follow‐up in 82.9% and 85.7% of LP and TP patients, respectively (*P >* 0.05). The AOFAS ankle‐hindfoot scale, Karlsson scale, FAAM, and LEFS scores did not differ significantly between groups at the final follow‐up (*P >* 0.05). The pre‐fracture and final postoperative scores on these four instruments did not differ significantly in the LP or TP group (*P >* 0.05). The mean times to radiographic union progression were 13.5 ± 7.1 weeks and 15.1 ± 10.2 weeks in the LP and TP groups, respectively (*P >* 0.05). The mean times to implant removal surgery reaffirming solid union were 15.6 ± 5.5 months and 14.8 ± 4.9 months in the LP and TP groups, respectively (*P >* 0.05). Hardware irritation was detected in five patients in the LP group (12.2%) and three in the TP group (7.1%) (*P >* 0.05). One patient in the LP group and two in the TP group developed superficial wound infections, which resolved without further surgical intervention.

**Conclusion:**

Conventional TP remains a good option for the fixation of Danis‐Weber type B distal fibular fractures, regardless of the biomechanical properties.

## Graphical abstract

For Danis‐Weber type B distal fibular fractures, fixation with conventional non‐locking one‐third tubular plates yielded clinical outcomes comparable to those of fixation with locking plates. Two Kirschner wires, instead of interfragmentary lag screws, were used for temporary fixation.

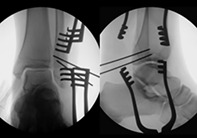

## Introduction

**Fig. 1:**
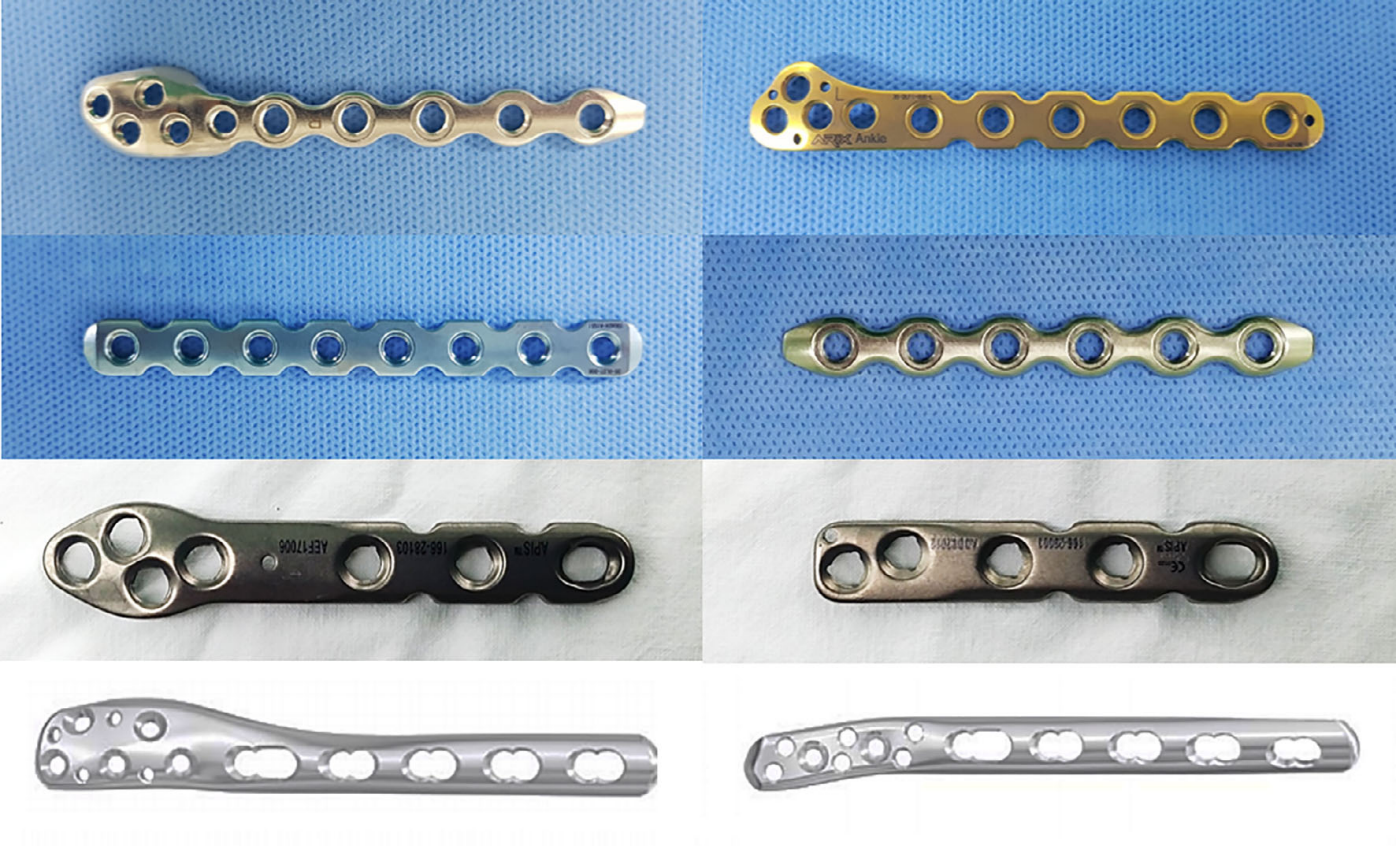
The various types of locking plate (LP) for fixing distal fibular fractures have been introduced into the orthopaedic implant market. The LP system has been developed in recent decades. The reason for the popularity of the LP system is based on previous studies demonstrating the biomechanical superiority of the LP design, especially in osteoporotic or comminuted periarticular fractures.

Ankle fracture is one of the most common injuries requiring surgical treatment^1^. Of the various types, distal fibular fractures are the most prevalent (57.6%)^2^. Danis‐Weber type B fractures are the most common form of distal fibular fractures; they are mainly caused by a supination‐external rotation injury and are characterized by an oblique fracture line^3^, ^4^. Of the various treatment options for distal fibular fractures^5^, ^6^, ^7^, ^8^, ^9^, ^10^, ^11^, ^12^, open reduction and internal fixation is the most preferred option to restore fibular length and rotation^13^, ^14^. Open reduction and internal fixation surgical methods for distal fibular fractures include lateral plating, posterior antiglide plating, and the lag screw‐only technique^10^, ^15^, ^16^. Conventional plating techniques depend on compression between the plate and cortical bone for stability. Fixation of a distal fibular fragment relies on unicortical cancellous bone to avoid intra‐articular penetration of the distal screws. The most difficult aspect is achieving adequate distal fixation in cases with a short segment or comminution of the distal fibula or osteoporotic bone. Various kinds of implants have been developed to compensate for this difficulty, including a locking plate (LP) system, tibial pro‐fibular screw^17^, Kirschner wire cage, and intramedullary fibular nails^18^, ^19^. Among them, the LP system has become popular in recent decades, and various types of LPs have been introduced into the orthopaedic implant market (Fig. 1). A LP may be advantageous for osteoporotic distal fibular fractures because its fixed‐angle construct precludes the need for contact or compression between the plate and bone, such that there is less reliance on bone mineral density (BMD) to stabilize the fracture^20^. Several cadaver studies have indicated that LP is more biomechanically advantageous than the conventional non‐locking one‐third tubular plate (TP) for fixing distal fibular fractures^20^, ^21^. According to Kim *et al*.^21^, the LP construct with two distal unicortical screws is mechanically equivalent to a standard plate with three distal screws in cadavers. Following a biomechanical report, the clinical use of LP fixation for distal fibular fractures has been increasing, along with its use for other anatomical sites, such as the proximal humerus, distal radius, distal femur, and proximal tibia, despite the cost of an LP implant being higher than that of a TP. Eckel *et al*.^22^ conducted a comparative biomechanical study to identify the plate types associated with improved distal fibular fracture outcomes. However, the authors concluded that their experimental results were insufficient to identify a superior plate type.

Some studies have reported clinical outcomes of distal fibular fractures treated with and without LPs^23^, ^24^, ^25^, ^26^, ^27^. However, the results were variable and there was heterogeneity in fracture classification and implant type. There is still a lack of clinical evidence in the literature regarding the most suitable plate type for distal fibular fractures, particularly Danis‐Weber type B fractures. Therefore, the purposes of this study are as follows: (i) to compare clinical outcomes between LP and TP fixation for Danis‐Weber type B distal fibular fractures; (ii) to provide guidance on plate selection for Danis‐Weber type B distal fibular fracture fixation.

## Methods and Materials

### Inclusion and Exclusion Criteria

The inclusion criteria were as follows: (i) a Danis‐Weber type B fibular fracture, with >2 mm displacement, shortening, or rotation; (ii) patients who underwent surgical fixation using either LP or TP system which was randomly selected by one surgeon (foot and ankle orthopaedic specialist); (iii) patients with clinical records as well as perioperative radiographs; (iv) a minimum 12‐months follow‐up.

The exclusion criteria were as follows: (i) open fractures; (ii) pilon fractures; (iii) concomitant ankle dislocation; (iv) bilateral ankle fractures; and (v) severe syndesmotic or medial injury that required additional transsyndesmotic screw fixation or deltoid ligament repair.

### Patients

This was a retrospective cohort study comparing clinical outcomes between LP and TP fixation for Danis‐Weber type B distal fibular fractures. Patients were enrolled consecutively from March 2013 to July 2018. All patients provided written informed consent before surgery, as well as consent for publication. All data were extracted from electronic medical records. This study was approved by the institutional review board of the corresponding author's university hospital (No. VC21RISI0182).

Finally, a total of 83 out of 132 patients treated with either LP or TP fixation were included. Patients were divided into two groups according to the type of implant used:

**LP group (n = 41)**: 2.7‐mm/3.5‐mm Locking Distal Fibular Plate (Arthrex, Inc., Naples, FL, USA).

**TP group (n = 42)**: 3.5‐mm/4.0‐mm Low Profile Third Tubular Plate (Arthrex, Inc., Naples, FL, USA).

### Surgical Procedures

All procedures were performed by the same surgeon who is the corresponding author of this article. The specific process is described in the following steps.

#### Anesthesia and Position

The patients were placed in the lateral recumbent position on a radiolucent operating table under general or spinal anesthesia. A pressure pneumatic tourniquet was placed at the proximal thigh, and inflated to 280 mmHg after exsanguination.

#### Approach, Exposure, Reduction, and Fixation Techniques

**Fig. 2:**
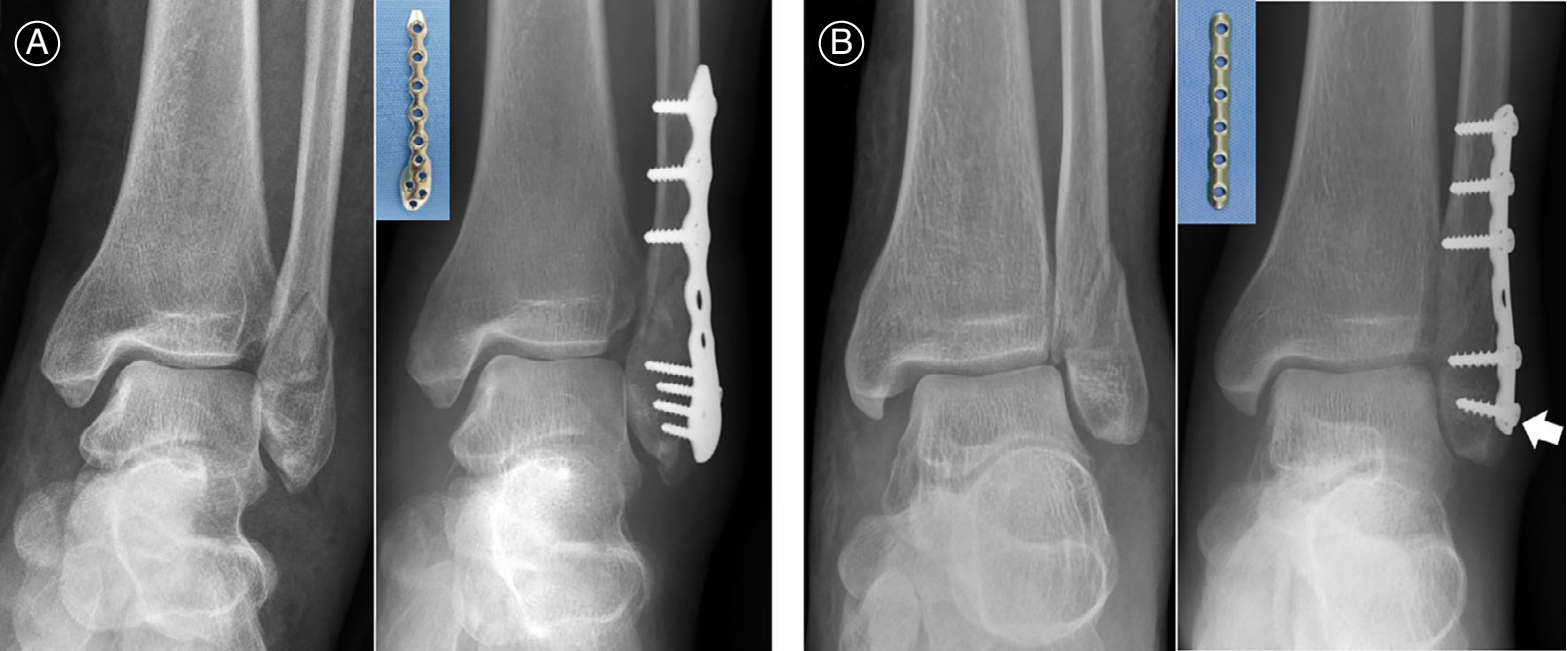
Radiographs of Danis‐Weber type B distal fibular fractures treated by plating osteosynthesis: (A) A 45‐year‐old female patient was treated with locking plate (LP) fixation. Three proximal 3.5‐mm bicortical non‐locking screws and four distal 2.7‐mm unicortical locking screws were used; (B) A 56‐year‐old male patient was treated with one‐third tubular plate (TP) fixation. Three 3.5‐mm bicortical non‐locking screws were used for proximal holes, while two 4.0‐mm unicortical cancellous screws were used for distal holes. Two technical tips are recommended to provide a good bone purchase of the distal fragment during TP fixation. First, the plate should be pre‐bent anatomically along the lateral aspect of distal fibula. Second, the most distal unicortical cancellous screw fixation should be slightly angled to prevent pulling out (white arrow).

All operations were performed through a lateral approach. After manual traction for fracture reduction, we compressed the fracture site using a reduction clamp, and fixed two Kirschner wires temporarily across the reduced oblique fracture, instead of using an interfragmentary lag screw; then, the LP or TP was placed on the lateral aspect of the distal fibula after bending. In the LP group, three proximal 3.5‐mm bicortical non‐locking screws and at least three distal 2.7‐mm unicortical locking screws were used (Fig. 2A). In the TP group, three proximal 3.5‐mm bicortical non‐locking screws and two distal 4.0‐mm unicortical cancellous screws were used. To provide good bone purchase of distal fragment in the TP group, we used “two technical tips” from AO surgery reference^28^. First, the TP should be pre‐bent anatomically along the lateral aspect of distal fibula. Second, the most distal unicortical cancellous screw fixation should be slightly angled to prevent pulling out (Fig. 2B). We removed the Kirschner wires after adequate plate fixation. Fracture reduction, stable fixation, and ankle joint congruency were confirmed during the operation using C‐arm imaging.

#### Incision Closure

A drainage tube was placed and the wound was closed in the same manner in both groups; the subcutaneous fascia was closed with synthetic absorbable interrupted sutures, and the skin was closed with a horizontal mattress pattern using non‐absorbable monofilament sutures.

### Postoperative Management

The ankle joint was maintained in a neutral position postoperatively using a short‐leg splint. From the second day after surgery, the patients started active ankle range of motion (ROM) exercises, then were allowed to walk with crutches without weight‐bearing for 2–4 weeks in a walking boot or splint. After 4–6 weeks, the patients were allowed to walk with partial weight‐bearing without a walking boot or splint and start passive ankle ROM exercises at the orthopaedic rehabilitation unit. After 8 weeks, the patients were allowed to walk with full weight‐bearing, and after 10–12 weeks they were allowed to run slowly on a flat floor. All sports activities, with progressively heavier loads, were allowed 4–6 months postoperatively after confirming bony union on plain radiographs.

### Data Collection and Assessments

#### Baseline Data

Demographic data of patients including age, gender, body mass index (BMI), diabetes mellitus (DM), and smoking history were collected. The duration of follow‐up was investigated in both groups. The proportion of comminuted fracture patterns in the two groups was investigated during surgery.

#### Range of Motion (ROM)

**Fig. 3:**
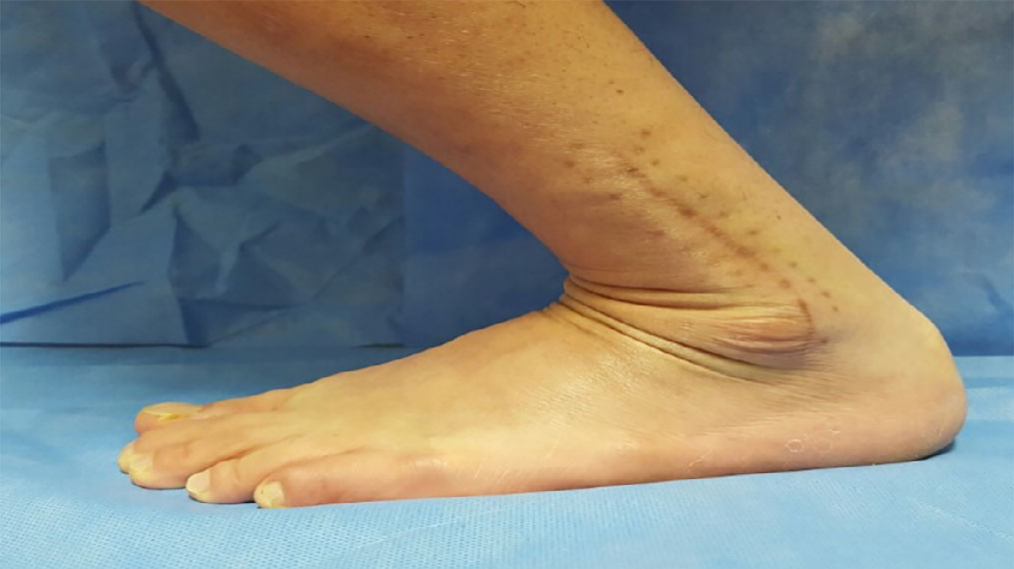
The ankle range of motion (ROM) at the final follow‐up visit was assessed. Maximal dorsiflexion ROM was measured with the forward bending posture in a standing position. Full recovery of dorsiflexion ROM was confirmed if patients could perform forward bending posture with similar degrees as much as their unaffected ankle or at least 10°–15° dorsiflexion.

The ankle ROM at the final follow‐up visit was also determined. The ROM of each ankle was measured as follows. Dorsiflexion was assessed in a standing position in terms of the patient's ability to perform forward bending (Fig. 3), and plantar flexion was assessed in a sitting position with the foot hanging freely over the end of an examination bed. The degree of plantar flexion was measured as far as the patient could reach with the help of the examiner, not with the help of gravity. The measuring device was a standard (30‐cm) goniometer, and two measurements were made per patient by the corresponding author. We obtained the mean value of those results. If the final ROM was below the normal side ankle ROM or at least text reference ROM (approximately 10°–15° of dorsiflexion to 45°–50° of plantar flexion)^29^, the patient was considered to have limited ROM.

#### Four Instruments to Assess Functional Ankle Joint Outcomes

We also obtained the American Orthopaedic Foot and Ankle Society (AOFAS) ankle‐hindfoot scale, Karlsson scale, Foot and Ankle Ability Measure (FAAM), and Lower Extremity Functional Scale (LEFS) scores. These four instruments were completed before surgery and at the final follow‐up visit, and the scores were compared.

##### American Orthopaedic Foot and Ankle Society (AOFAS) Ankle‐Hindfoot Scale

The AOFAS ankle‐hindfoot scale^30^ is among the most commonly used instruments for measuring outcome of treatment in patients who sustained a complex ankle or hindfoot injury. This scale grades ankle, subtalar, talonavicular, and calcaneocuboid joint levels. It consists of a patient‐reported and a physician‐reported part. The AOFAS scale is a 100‐point score system with three categories: 40 points to pain, 50 points to function, and 10 points to alignment. A total score of 100 points is possible in a patient with no pain, full range of sagittal and hindfoot motion, no ankle or hindfoot instability, good alignment, ability to walk more than six blocks, ability to ambulate on any walking surface, no limp, no limitation of daily or recreational activities, and no assistive devices needed for ambulation.

##### Karlsson Scale

The Karlsson scale^31^ consists of eight items. These items are a subjective evaluation of functional stability, pain, swelling, and stiffness. Tasks of daily life, such as running and stair climbing, working ability, as well as sports activity and leisure activities are evaluated. The maximum score is 100 points and a higher score represents a higher level of ankle function. Subjective or functional stability is given 25 points. Absence of pain is given 20 points. Swelling is given 10 points. Stiffness is given 5 points. Activities such as running and stair climbing are given a score of 10 points each. Overall assessment of work, sports activities, and leisure time activities is given a score of 15 points in combination. The last item, the need for wearing an ankle support, is given a score of 5 points. The Karlsson scale scoring was originally developed to assess ankle joint function after ligament reconstruction for chronic lateral instability. However, it can also be used to evaluate ankle joint function before and after treatment for injury. The Karlsson scale does not only relate to symptoms, but also to the functional level of the individual.

##### Foot and Ankle Ability Measure (FAAM)

The FAAM^32^ was developed to meet the need for a self‐reported evaluative instrument that comprehensively assesses physical function of individuals with musculoskeletal disorders of the leg, foot, and ankle. The FAAM is a reliable, valid, and responsive measure of patient‐reported outcome for individuals participating in physical therapy, with or without operative intervention. The FAAM consists of 21 questions indicating Activities of Daily Living (ADL) subscale. A second section of the FAAM consists of eight questions indicating Sports subscale. FAAM scores are converted to percentages for clinical use.

##### Lower Extremity Functional Scale (LEFS)

The LEFS^33^ is easy to administer and score and is applicable to a wide range of disability levels and conditions and all lower‐extremity sites. It is more interpretable with respect to understanding error associated measurement and for determining minimally clinically important score changes and is a sufficient measure of reliability, validity, and sensitivity to change, at a level that is commensurate with utilization at an individual patient level. The LEFS can be used by clinicians as a measure of patients' initial function, ongoing progress, and outcome as well as to set functional goals. The LEFS consists of 20 items, with scores ranging from 0 (extreme difficulty/unable to perform activity) to 4 (no difficulty). The total score can be obtained by summing the scores of the individual items. The maximum score of 80 indicates no functional limitations and the minimum score of 0 indicates extreme limitations.

#### Radiographic Assessment for Time to Fracture Union

The progression of radiographic union was evaluated. All pre‐ and postoperative radiographs of the ankles were assessed by two independent observers, and the interval to radiographic union was noted. If no consensus was reached, the senior author made the final decision. The progression of union was defined as evidence of bridging bone in three of four cortices in the anteroposterior and lateral planes on simple radiographs^23^, ^34^.

#### Intraoperative Assessment for Fracture Union Completion

The implant removal time was investigated. We reaffirmed the “solid” union intraoperatively at the time of implant removal.

#### Postoperative Complications

Complications related mainly to the surgical site were investigated.

### Statistical Analysis

For continuous variables, the student's *t*‐test was used for the comparison of mean age, BMI, and the four instrument scores at the final follow‐up between the two groups. The paired *t*‐test was used to compare pre‐fracture and postoperative (final visit) scores on the four instruments detailed above in the same patient. The Wilcoxon rank sum test with continuity correction was used for the comparison of mean follow‐up durations and mean times to fracture union between the two groups.

For categorical variables, the chi‐square test was used for comparisons of gender distribution, DM and smoking history, the proportion of comminuted fracture and the ankle ROM at the final follow‐up visit between the two groups. The Fisher's exact test was used for the comparison of postoperative complications. Statistical analyses were performed using R software (ver. 3.5.3; R Development Core Team, R Foundation for Statistical Computing, Vienna, Austria). The mean and standard deviation were computed for normally distributed continuous variables, and the median and interquartile range (25th–75th percentile) for non‐normally distributed continuous data. A *P*‐value <0.05 was considered statistically significant.

## Results

### General Results

As summarized in Table 1, the mean ages were 53.3 ± 17.5 years (range, 16–80 years) in the LP group and 47.6 ± 17.0 years (range, 14–68 years) in the TP group (*P* = 0.397). Of the 83 patients, 44 were male (53.0%) and 39 were female (47.0%); the gender distribution was not significantly different between the two groups (*P* = 0.399). The mean BMI were 23.8 ± 3.4 kg/m^2^ (range, 18.1–29.8 kg/m^2^) in the LP group and 24.5 ± 3.7 kg/m^2^ (range, 18.2–34.1 kg/m^2^) in the TP group (*P* = 0.375). Five of 41 patients (12.2%) in the LP group and 6 of 42 patients (14.3%) in the TP group (*P* = 0.299) had DM. Thirteen of 41 patients (31.7%) in the LP group and 12 of 42 patients (28.6%) in the TP group (*P* = 0.892) had a smoking history. The mean follow‐up durations were 16.8 ± 7.7 months (range, 13.0–19.0 months) in the LP group and 16.1 ± 6.2 months (range, 12.0–20.0 months) in the TP group (*P* = 0.905). Intraoperatively, 18 of 41 patients (43.9%) in the LP group and 10 of 42 patients (23.8%) in the TP group (*P* = 0.088) had comminuted fracture patterns.

**TABLE 1:** Demographic and clinical data between the locking plate and non‐locking one‐third tubular plate groups

	LP (n = 41)	TP (n = 42)	*P*‐value
Mean age (years)	53.3 ± 17.5	47.6 ± 17.0	0.397^†^
Gender			
Male	24 (58.5%)	20 (47.6%)	0.399^††^
Female	17 (41.5%)	22 (52.4%)	
Mean BMI (kg/m^2^)	23.8 ± 3.4	24.5 ± 3.7	0.375^†^
Diabetes mellitus	5 (12.2%)	6 (14.3%)	0.299^††^
Smoking history	13 (31.7%)	12 (28.6%)	0.892^††^
Proportion of comminuted fracture	18 (43.9%)	10 (23.8%)	0.088^††^
Range of motion			
Full	34 (82.9%)	36 (85.7%)	0.962^††^
Limited	7 (17.1%)	6 (14.3%)	

BMI, body mass index; LP, locking plate; TP, non‐locking one‐third tubular plate.

^**†**^ The *P*‐value was calculated using a Student's *t*‐test; not statistically significant between the two groups.

^**††**^ The *P*‐value was calculated using a chi‐square test; not statistically significant between the two groups.

### Comparison of Clinical Outcomes

#### Range of Motion (ROM)

The proportions of patients who achieved full ROM at the final follow‐up visit were 82.9% (34 of 41 patients) in the LP group and 85.7% (36 of 42 patients) in the TP group (*P* = 0.962) (Table 1).

#### Four Instruments to Assess Functional Ankle Joint Outcomes

The AOFAS ankle‐hindfoot score was not significantly different between the LP (83.9 ± 14.3) and TP (89.4 ± 9.5) groups (*P* = 0.253) at the final follow‐up. The Karlsson score was not significantly different between the LP (69.7 ± 18.8) and TP (82.5 ± 17.5) groups (*P* = 0.077) at the final follow‐up. The FAAM, which comprises ADL and Sports subscales, was not significantly different between the two groups at the final follow‐up (ADL: LP, 95.1% [84.2; 97.5] *vs* TP, 95.1% [83.9; 97.6], *P* = 0.982; Sports: LP, 80.7% [60.0; 94.3] *vs* TP, 82.9% [72.9; 93.6], *P* = 0.818). The LEFS score was not significantly different between the LP (66.0 [51.0; 76.5]) and TP (76.0 [69.0; 80.0]) groups (*P* = 0.210) at the final follow‐up. As summarized in Table 2, the pre‐fracture scores on the four instruments were compared with those at the final postoperative follow‐up visit in each group; there was no significant difference of the AOFAS ankle‐hindfoot score in the LP (*P* = 0.137) or TP (*P* = 0.481) groups; the Karlsson score in the LP (*P* = 0.159) or TP (*P* = 0.398) groups; the FAAM‐ADL in the LP (*P* = 0.375) or TP (*P* = 0.466) group; the FAAM‐Sports in the LP (*P* = 0.229) or TP (*P* = 0.535) groups; the LEFS in the LP (*P* = 0.228) or TP (*P* = 0.910) groups.

**TABLE 2:** Comparison of four instruments to assess functional outcomes between patients' usual activities (pre‐fracture state) and final postoperative follow‐up visit within the locking plate or non‐locking one‐third tubular plate groups

	LP			TP		
	Usual	Final follow‐up	*P*‐value	Usual	Final follow‐up	*P*‐value
AOFAS ankle‐hindfoot scale	99.0 [81.5;100.0]^‡^	88.0 [75.0; 95.0]	0.137^§^	99.0 [82.0;100.0]	90.0 [85.0;98.0]	0.481^§^
Karlsson scale	81.2 ± 22.1^‡^	69.7 ± 18.8	0.159^§^	97.5 [68.5;100.0]	87.0 [82.0;92.0]	0.398^§^
FAAM‐ ADL (%)	96.1 [87.1;100.0]	95.1 [84.2;97.5]	0.375^§^	98.3 [75.1;100.0]	95.1 [83.9;97.6]	0.466^§^
FAAM‐ Sports (%)	88.2 [78.9;100.0]	80.7 [60.0;94.3]	0.229^§^	95.7 [58.2;100.0]	82.9 [72.9;93.6]	0.535^§^
LEFS	74.0 [67.0;80.0]	66.0 [51.0;76.5]	0.228^§^	79.5 [54.0;80.0]	76.0 [69.0;80.0]	0.910^§^

ADL, Activities of Daily Living; AOFAS, American Orthopaedic Foot and Ankle Society; FAAM, Foot and Ankle Ability Measure; LEFS, Lower Extremity Functional Scale; LP, locking plate; TP, non‐locking one‐third tubular plate.

^‡^ The mean and standard deviation were computed for normally distributed continuous variables, whereas the median and interquartile range (IQR) [25th to 75th percentile] were calculated for non‐normally distributed continuous data. The *P*‐value was calculated using a paired *t*‐test.

^§^ not statistically significant between pre‐fracture state and final postoperative follow‐up visit in both groups.

#### Times to Fracture Union

The mean time to radiographic union progression was 13.5 ± 7.1 weeks in the LP group and 15.1 ± 10.2 weeks in the TP group (*P* = 0.717). The mean time to implant removal surgery reaffirming intraoperative solid union was 15.6 ± 5.5 months in the LP group and 14.8 ± 4.9 months in the TP group (*P* = 0.728).

#### Postoperative Complications

Hardware irritation was detected in five of 41 patients (12.2%) in the LP group and three of 42 patients (7.1%) in the TP group (*P* = 0.483). One of 41 patients (2.4%) in the LP group and two of 42 patients (4.8%) in the TP group (*P* = 1.000) developed a superficial wound infection, which was controlled by intravenous antibiotics and meticulous wound management without further intervention. No patient in either group required removal of an implant before complete fracture union because of hardware irritation or infection. No other complications were encountered, such as nonunion, malunion, or neurovascular injury.

## Discussion

### Main Findings

We retrospectively assessed the medical records of patients treated using plate fixation for Danis‐Weber B distal fibular fractures, which is the most common clinical pattern. We hypothesized that LP fixation would provide better clinical outcomes due to its enhanced biomechanical stability relative to the TP. However, we did not observe any significant difference between the LP and TP groups in demographic or clinical data, including proportion of comminuted fracture, ankle ROM, scores on the AOFAS ankle‐hindfoot scale, Karlsson scale, FAAM, and LEFS, time to fracture union, or postoperative complications.

### Comparison of Clinical Outcomes between LP and TP Fixation

Plate selection for distal fibular fractures remains controversial among clinicians; some favor the newer LP and others favor a conventional TP or other non‐LP. Although many biomechanical and clinical comparative studies have been reported, there remains a paucity of guidelines for clinicians to follow regarding plate selection to fix a distal fibular fracture. The LPs used for periarticular fractures have evolved steadily over the past decades. However, despite several cadaver studies demonstrating the biomechanical superiority of the LP design for cases of osteoporosis or comminuted fractures^20^, ^21^, ^35^, the clinical benefits of the LP have not been established. The *in vivo* (patient) environment is more suitable than the *in vitro* (cadaver) environment for assessing clinical outcomes. In our study, there were no significant differences between the LP and TP groups in terms of comparing clinical outcomes.

A biomechanical study by Eckel *et al*.^22^ using the Weber B fracture model to compare LPs and non‐LPs suggested that *in vivo* loading magnitudes are unknown and will vary depending on patient compliance, weight, and activity. Nguyentat *et al*.^36^ compared the biomechanical properties of LP and TP fixation for the AO Foundation/Orthopaedic Trauma Association (AO/OTA) 44‐B3.3 distal fibular fractures in cases of trimalleolar ankle injury. They suggested that a distal fibular LP does not provide a mechanical advantage for trimalleolar ankle injuries in individuals with normal BMD and an absence of fracture comminution. Zahn *et al*.^20^ and Kim *et al*.^21^ reported that the biomechanical attributes of LP are independent of BMD, such that it is a more functional treatment for osteoporotic and comminuted distal fibular fractures. However, the BMD was measured at different sites among cadaver studies, including the tibia, fibula, distal metaphysis, diaphysis, and even the calcaneus or proximal femur. This makes direct comparison of the results of these studies difficult. Also, it is difficult to model comminuted fractures that mimic those *in vivo* in cadaver specimens. Davis *et al*.^37^ questioned a previous cadaver study by Kim *et al*.^21^ comparing TPs with LPs. They concluded that the LP does not outperform its non‐locking counterparts, and that TPs provide adequate fixation for AO/OTA 44‐B2.1 fractures.

Several clinical studies have compared surgical outcomes between LPs and non‐LPs. Huang *et al*.^25^ reviewed 147 patients divided into TP, locking compression metaphyseal plate, and locking anatomical distal fibular plate groups. A Danis‐Weber subgroup was also analyzed and compared. No significant differences were observed among the three plates in the Weber C subgroup in terms of ankle ROM, radiographic reduction accuracy, complication rate, Olerud and Molander score, or the AOFAS scale score. In contrast, the LP groups showed better functional scores than the TP group in the Weber A and B subgroups. Tsukada *et al*.^38^ conducted a randomized controlled trial comparing the clinical effectiveness of locking and non‐locking neutralization plates for AO/OTA 44‐B fractures. They observed no differences in terms of the radiographic fracture union rate, 36‐Item Short Form Survey score, or wound complication rate between the plate groups. These results were consistent with our study, in which no clinical advantage of the LP over the TP was seen. We investigated the proportion of comminuted fracture, ankle ROM recovery, and four instruments of functional scores as clinical outcomes, and observed no significant group difference. The radiographic fracture union outcomes and postoperative complications were also not different between the groups, similar to other clinical comparative studies^25^, ^27^, ^38^, ^39^.

Patient‐reported outcome measurements (PROMs) are considered to be more important than physician‐reported outcome measurements. In clinical studies of orthopaedic surgery, many types of validated PROMs are increasingly being used to better understand the effectiveness of surgical treatment. In the current study, we used not only the physician‐reported AOFAS scale, but also three PROMs, namely the Karlsson scale, FAAM, and LEFS, to assess functional ankle joint outcomes in a comprehensive manner. Moreover, we compared pre‐fracture and postoperative (at the final follow‐up visit) scores on the four instruments. Although the pre‐fracture scores on all four instruments were numerically higher, the differences were not significant in either group. Therefore, the functional performance of patients with Danis‐Weber B distal fibular fractures can be expected to recover well regardless of whether LP or TP fixation is used.

### Technical Tips for TP Fixation to Obtain Comparable Outcomes to LP Fixation

In our study, we did not use interfragmentary lag screws. A recent prospective randomized study demonstrated that interfragmentary lag screws are not essential for precontoured lateral LP fixation in non‐comminuted supination‐external rotation lateral malleolar fractures, provided that intraoperative compression with a reduction clamp is performed^40^. The lag screw head and purchase impede proper plate placement. Hence, temporary fixation in our study involved two Kirschner wires instead of interfragmentary lag screws. In both groups, plate fixation was performed under compression of the fracture site using a reduction clamp in combination with temporary Kirschner wire fixation. Moreover, we did not follow the biomechanical report of Kim *et al*.^21^, and used only two distal unicortical cancellous screws in the TP group. We obtained comparable surgical outcomes in the LP and TP groups, and found that TP construct with two distal unicortical screws was clinically acceptable. We attribute these findings to the successful placement of the reduction clamp and Kirschner wires, which function to compress the fracture and maintain this compression, along with our “two technical tips” for TP fixation described above.

### Other Considerations for Clinical Comparisons between LP and TP

Although not assessed in our study, the cost‐effectiveness of a LP should be considered. The cost may be high depending on the surgery time, complications, length of hospital stay, and insurance system (which differs among countries), but newer LPs are generally more expensive than conventional plates. Moss *et al*.^41^ demonstrated that treating Weber B distal fibular fractures with a TP can be significantly less expensive than using a contoured LP, without any increase in the likelihood of complications, failure, or loss of reduction. Nguyentat *et al*.^36^ reported that overuse of LPs can be detrimental to healthcare systems, by consuming resources that could have been better used elsewhere. The cost issue has also been emphasized in cases of fracture at other anatomical sites^42^.

### Limitation of the Study

This study had some limitations that should be considered. First, it used a non‐randomized, retrospective design. Second, the bone density of the patients was not measured. Our cohort included young patients, and we are unable to routinely perform dual‐energy X‐ray absorptiometry scans for patients aged <65 years who use the national insurance system. Third, intra‐ and inter‐observer reliability were not evaluated for the ankle ROM and radiographic union assessments. Further randomized controlled trials with larger sample sizes or meta‐analyses are required to verify which plate is superior in various clinical conditions and patient populations.

### Conclusion

In conclusion, the LP is not a prerequisite for fixing Danis‐Weber type B distal fibular fractures, especially when the fracture pattern is not complicated. Surgeons can choose a TP at their discretion and still achieve a good clinical outcome. The conventional TP remains a good option for the fixation of Danis‐Weber type B distal fibular fractures, regardless of the biomechanical properties.
